# Dietary *Pediococcus acidilactici* supplementation enhances sperm quality and testicular function in roosters

**DOI:** 10.1016/j.psj.2025.105432

**Published:** 2025-06-21

**Authors:** Yanqiu Lv, Yinan Wang, Xuan Chen, Biao Xuan, Longzheng Yu, Wanfeng Liang, Yi Jin

**Affiliations:** Agricultural College of Yanbian University, Gongyuan Street, Yanji, Jilin 133002, PR China

**Keywords:** *Pediococcus acidilactici*, Reproductive parameters, RNA-seq, Roosters, Testicular histomorpholog

## Abstract

The ban on the use of antibiotics as growth promoters due to resistance issues has urged scientists to find alternatives to antibiotics. In recent years, probiotics have gained widespread attention for their potential benefits on systemic physiological functions. However, little is known about how *Pediococcus acidilactici* supplementation specifically influences sperm quality, testicular development, and the molecular mechanisms underlying male reproductive performance in roosters. Therefore, this study aimed to evaluate the effects of different levels of *Pediococcus acidilactici* supplementation on the reproductive performance of roosters. Roosters were randomly assigned to four groups: a control group (fed a basal diet) and three treatment groups supplemented with *Pediococcus acidilactici* at P1 (1 × 10^8^), P2 (1 × 10^9^), and P3 (1 × 10^10^) CFU/mL. Over a five-week trial period, semen parameters, motility, viability, mitochondrial membrane potential, apoptosis, protamine deficiency, reproductive hormone levels, testicular histological parameters, and transcriptomic profiles were assessed. The results demonstrated that the beneficial effects of *Pediococcus acidilactici* supplementation on rooster semen quality progressively increased with prolonged feeding duration. Notably, in the final week, compared with the control group, the P2 and P3 groups exhibited significantly improved sperm parameters (*P* < 0.01), motility (*P* < 0.05), viability, and mitochondrial function (*P* < 0.01), while apoptosis (*P* < 0.05) and protamine deficiency (*P* < 0.01) were significantly reduced. Additionally, reproductive hormone levels, testicular histology, and testicular antioxidant capacity were also significantly improved in the P2 and P3 groups (*P* < 0.05). Finally, transcriptomic analysis identified five key genes (RAF1, PIK3R1, ATRX, ARID4A, and SOX9) and four signaling pathways involved in testicular development. These findings suggest that supplementation with an appropriate level of *Pediococcus acidilactici*, particularly at 1 × 10^9^ CFU/mL, probably can enhance the reproductive capacity of roosters by improving sperm quality and testicular development.

## Introduction

Livestock production in general and domestic chicken production in particular plays a vital socio-economic role for people living in low-income countries of Africa and Asia (Mohamadinejad et al., 2024). Domestic chickens are widely distributed avian species around the world, due to their short generation interval and adaptability in a wide range of agro ecologies (Khabiri et al., 2025). The domestic chickens provide high quality protein and income for the poor rural households and are the most widely kept livestock species in the world (Mohamadinejad et al., 2024; Mohammadabadi et al., 2010). This is due to the presence of the valuable traits of chicken like disease resistance, adaptation to harsh environments and ability to utilize poor quality feeds (Khabiri et al., 2023; Shahdadnejad et al., 2016). The quality of semen in breeding roosters is a critical factor influencing productivity and economic efficiency in the poultry industry. High-quality semen not only enhances fertilization rates but also reduces the frequency and cost of artificial insemination. In recent years, the use of probiotics as feed supplements in animal production has increased considerably due to the ban on antibiotic growth promoters in livestock (Kober et al., 2022). Simultaneously, probiotics have garnered widespread attention for their beneficial effects on gut health (Barigela and Bhukya, 2021; Kim et al., 2019; Sun et al., 2024; Wang et al., 2017), immune function (Arani et al., 2021; Izquierdo et al., 2024; Woo et al., 2024), and systemic physiology. Li et al. identified *Pediococcus acidilactici* LC-9-1 as a potential poultry probiotic with antibacterial and antioxidant properties (Li et al., 2023). Additionally, certain probiotics or specific bacterial strains can produce antioxidants such as glutathione and superoxide dismutase, which play a crucial role in generating healthy sperm and maintaining sperm quality to ensure motility, energy acquisition, and DNA integrity, thereby protecting sperm from oxidative damage (Fernandez and O'Flaherty, 2018; Fernandez et al., 2019). Among probiotics, *Pediococcus acidilactici* is a lactic acid bacterium that has shown strong potential in regulating broiler growth performance due to its ability to enhance nutrient absorption, reduce oxidative stress, and modulate systemic inflammation (Al-Emran et al., 2022; Jeong et al., 2020; Li et al., 2023; Meng et al., 2010; Mikulski et al., 2012).

While existing studies have explored the effects of probiotics on general poultry health and growth performance, their specific roles in reproductive parameters, particularly in roosters, remain underexplored. Recent research has highlighted the critical relationship between sperm quality, mitochondrial function, and testicular health in determining male fertility (Gao et al., 2022; Ghadimi et al., 2024; Hong et al., 2022). Moreover, advancements in transcriptomic analysis have provided novel insights into the molecular mechanisms underlying testicular development and sperm production (Hong et al., 2022), offering opportunities to identify key regulatory pathways influenced by dietary interventions. However, there is still a lack of understanding regarding how specific probiotics, such as *Pediococcus acidilactici*, influence these complex processes. Additionally, the molecular mechanisms through which *Pediococcus acidilactici* affects testicular development and function have not been elucidated.

In summary, this study aims to address these gaps by systematically investigating the effects of different levels of *Pediococcus acidilactici* supplementation on sperm quality, testicular histology, reproductive hormone levels, and transcriptomic profiles in roosters. To the best of our knowledge, this is the first study to comprehensively assess the reproductive effects of *Pediococcus acidilactici* in roosters using a multi-level approach. By integrating physiological, biochemical, and transcriptomic analyses, we aim to provide new insights into the role of probiotics in enhancing male fertility and to identify potential molecular targets for improving reproductive performance in poultry.

## Materials and methods

### Experimental animals, design, and diet management

All animal experiments followed established protocols and were approved by the Experimental Animal Ethics Committee of Yanbian University, China (Approval No. SYXK2020-0009). *Pediococcus acidilactici* was isolated and stored by the School of Animal Medicine at Yanbian University. The yellow-feathered roosters used in this study were obtained from Yanbian Lire Livestock Technology Co., Ltd. Twenty hundred mature yellow-feathered roosters were carefully selected from a commercial flock at 43 weeks of age. The ambient temperature was maintained at 22 ± 2 °C, and the lighting regimen consisted of 14 h of light and 10 h of darkness per day. Prior to the experiment, the roosters were provided with a basal diet devoid of *Pediococcus acidilactici*. The composition of the basal diet followed the nutrient requirements outlined by the National Research Council (1994, Table 1). To familiarize the roosters with the semen collection procedure, a two-week adaptation period was conducted at 44 and 45 weeks of age, following the method described by Mehdipour et al. (2018). At the end of the adaptation period, when the roosters reached 46 weeks of age, they were randomly divided into four treatment groups, each consisting of five roosters. The treatments were as follows: a control group (basal diet supplemented with sterile normal saline), P1 group (basal diet supplemented with *Pediococcus acidilactici* at 1 × 10^8^ CFU/mL), P2 group (basal diet supplemented with *Pediococcus acidilactici* at 1 × 10^9^ CFU/mL), and P3 group (basal diet supplemented with *Pediococcus acidilactici* at 1 × 10^10^ CFU/mL). All roosters were housed in wire cages under temperature-controlled conditions and were provided ad libitum access to diet and water.

**Table 1:** Ingredient composition and calculation of ingredients in a basal rooster diet.

Ingredient	Content
Corn	55.86
Soybean meal	19.10
Soybean oil	4.12
Wheat bran	15.12
NaCl	0.35
DL-methionine	0.15
Limestone	3.50
Dicalcium phosphate	1.52
aTrace mineral premix	0.30
bVitamin premix	0.02
Choline chloride (50 %)	0.23
Total	100
Calculation of nutrients	
Metabolizable energy(MC/kg)	12.05
Crude protein	15.40
Calcium	1.05
Methionine	0.38
Lysine	1.15
Available phosphorus	0.41

a Trace mineral premix provided the follow per kg of basal diet: Cu, 8 mg; Zn, 75 mg; Fe, 80 mg; Mn, 100 mg; Se, 0.15 mg; I, 0.35 mg.

b Vitamin premix provided the follow per kg of basal diet: vitamin A, 125 000 IU; vitamin D3, 2 500 IU; vitamin E, 30 IU; vitamin K3, 2.65 mg; thiamine, 2 mg; riboflavin, 6 mg;vitamin B12, 0.025 mg; biotin, 0.0325 mg; folic acid, 1.25 mg; pantothenic acid, 12 mg; nicotinic acid, 50 mg.

### Semen sample collection and quality assessment

Semen samples from twenty roosters were collected three times per week for five consecutive weeks using the dorsal-abdominal massage method. Samples from all roosters were immediately transferred to centrifuge tubes and placed in a 37 °C incubator for sperm quality evaluation. Sperm motility and motion parameters were measured using the HTM-IVOS II system (Hamilton Thorne Biosciences, Beverly, MA, USA). Each semen sample was repeated three times. Briefly, 3 µL of diluted semen was loaded into a pre-warmed chamber for analysis. Each assessment involved randomly selecting a minimum of 8 fields for parameter evaluation (Hong et al., 2022; Mehdipour et al., 2020), including sperm motility ( %), linearity index (%), straightness index (%), average path velocity (µm/s), straight line velocity (µm/s), and curvilinear velocity (µm/s).

### Evaluation of sperm membrane integrity

To evaluate sperm membrane functionality, the hypo-osmotic swelling test (HOST) was performed (Mehdipour et al., 2022). A 5 µL aliquot of diluted semen was mixed with 50 µL of hypo-osmotic swelling solution (13.512 g/L fructose and 7.352 g/L sodium citrate) and incubated at 37 °C for 30 min. After incubation, a smear of the semen sample was prepared on a glass slide, covered with a coverslip, and examined under a phase-contrast microscope at 400 × magnification (Ts2, Nikon, Japan). For each sample, at least 200 spermatozoa were counted. Sperm exhibiting tail curling were classified as having intact membrane functionality.

### Evaluation of sperm viability and mitochondrial membrane potential (MMP)

Sperm from each group were analyzed using combined Hoechst 33342/PI/JC-1 staining to evaluate sperm viability (head) and MMP (midpiece). A 500 µL aliquot of sperm sample, pre-warmed to 37 °C, was mixed with 2.5 µL of 20 µmol/L Hoechst 33342 dissolved in dimethyl sulfoxide (DMSO) and 5 µL of 153 µmol/L JC-1 in DMSO (Han et al., 2019). The mixture was incubated in a water bath at 37 °C in the dark for 20 min. Afterward, 2.5 µL of 2.4 mmol/L PI staining solution was added, thoroughly mixed, and further incubated under the same conditions for an additional 10 min. Stained sperm samples were observed under a fluorescence microscope (IX71, Olympus, Japan), and at least 200 spermatozoa per sample were evaluated. Sperm classification criteria were as follows: sperm with blue fluorescence in the head were considered viable, while those with red fluorescence in the head were considered non-viable. Sperm exhibiting red fluorescence in the midpiece were classified as having high MMP, whereas those with green or no fluorescence in the midpiece were classified as having low MMP.

### Assessment of sperm protamine deficiency via CMA3 staining

Each semen sample was washed twice in PBS solution (without Ca^2+^ and Mg^2+^) and then smeared onto glass slides using a pipette. The slides were fixed with lignin solution (methanol:acetic acid, 3:1) at 4 °C for 5 min. After fixation, 100 µL of CMA3 staining solution was applied to each slide, and the samples were incubated in the dark for 20 min. Following staining, the slides were rinsed in McIlvain buffer and air-dried. Stained sperm were examined under a fluorescence microscope (Ts2, Nikon, Japan), with at least 200 spermatozoa assessed per sample. Interpretation criteria: sperm heads exhibiting bright green fluorescence were classified as CMA3-positive, indicating abnormal chromatin structure and DNA damage, whereas those with dim green fluorescence were classified as CMA3-negative, indicating normal chromatin structure and intact DNA.

### RNA Extraction and qRT-PCR Analysis

Total RNA was extracted from semen samples of each rooster using the TRNzol Universal Total RNA Extraction Kit (DP424, Tiangen, Beijing, China). The extracted RNA was reverse-transcribed into cDNA using the FastKing One-Step Genomic DNA Removal and cDNA Synthesis Kit (KR118, Tiangen, Beijing, China). The reaction mixture (20 µL) consisted of 4 µL of 5 × FastKing-RT SuperMix, 2 µL of template RNA, and 14 µL of RNase-free ddH_2_O. The reverse transcription was performed at 42 °C for 15 min. The expression of the *CASPASE3* and *BCL-2* genes was analyzed using qRT-PCR with specific primers. RT-qPCR was conducted using the SuperReal PreMix Plus (SYBR Green) and the PCRmax Eco 48 Real-Time PCR System. Primer sequences were designed based on the study by Najafi et al. (2021), with GAPDH used as the internal reference gene. The specific primers for *CASPASE3* and *BCL-2* are listed in Table 2. All primers were synthesized by Sangon Biotech Co., Ltd. (Shanghai, China).

**Table 2:** Primer sequences used for the quantitative real-time PCR.

Genes	Primer Sequence (5′– 3′)	Tm	Product Size (bp)	Accession Number
*BCL-2*	F: AACATTGCCACCTGGATGAC R: CGAACAAAGGCTCATACTGT	55.59 52.20	118	NM_205339.2
*CASPASE3*	F: AACCAGCCTTTTCAGAGGTGAC R: CTGGTCCACTGTCTGCTTCAATA	57.55 56.71	119	NM_204725.1
GAPDH	F: TGATGCCCCCATGTTTGTGA R: TGGCATGGACAGTGGTCATA	57.62 58.74	164	NM_204305.1

### Assessment of the serum hormone levels

At week 5, 2 mL of blood was collected from the brachial vein of each rooster using a vacuum collection tube. The samples were centrifuged at 3,000 r/min for 10 min at 4 °C to separate the serum. Serum concentrations of luteinizing hormone (LH, HY-10025T), follicle-stimulating hormone (FSH, HY-10024T), and testosterone (T, HY-C0005) were determined using a commercial Enzyme-Linked Immunosorbent Assay (ELISA) kit (Beijing Sino-UK Institute of Biological Technology, Beijing, China) following the manufacturer’s instructions. All assays were conducted in 96-well plates, and absorbance was measured at 450 nm with a microplate reader (DR-200BS, Wuxi Hiwell-Diatek Instruments Co., Ltd., Wuxi, China).

### Antioxidant indices assays

Following the methodology described by Long et al. (2022), testicular total superoxide dismutase (T-SOD, A001), glutathione peroxidase (GSH-Px, A005), total antioxidant capacity (T-AOC, A015), and malondialdehyde (MDA, A003) levels were measured using a microplate reader (Thermo Fisher Scientific, MA, USA) according to the instructions provided with the respective assay kits. All assay kits were purchased from Nanjing Jiancheng Bioengineering Institute (Nanjing, China).

### Evaluation of testicular morphology and weight

During the final week of the feeding trial, testes were collected and weighed. The left testis was sectioned into small pieces (1.0 cm × 1.0 cm × 0.5 cm) and fixed in 10 % neutral buffered formalin for 48 hours. Fixed samples were embedded in paraffin, sectioned into 5-µm slices, and stained with hematoxylin and eosin. For each rooster, five representative microscopic fields were randomly selected and observed under a light microscope at 20 × magnification for histological analysis. The diameter of the seminiferous tubules, seminiferous epithelial thickness were measured using Image-Pro Plus software (version 6.0, Media Cybernetics, Inc., USA). The right testis was rapidly frozen in liquid nitrogen and stored at –80 °C for further analysis.

### RNA-Seq analysis

Total RNA was extracted from seven testes (three from the control group and four from the P2 group) using the TRIzol method (Invitrogen, CA, USA) and treated with RNase-free DNase I (Takara, Kusatsu, Japan). RNA degradation and contamination were assessed using 1 % agarose gels. RNA concentration was measured with an Agilent 2100 Bioanalyzer (Agilent Technologies, CA, USA), and its quality and integrity were evaluated using a NanoDrop spectrophotometer (Thermo Scientific, DE, USA). Sequencing libraries were generated using the NEBNext Ultra^TM^ RNA Library Prep Kit for Illumina (NEB, USA) according to the manufacturer's recommendations, and index sequences were added to attribute sequences to each sample. RNA sequencing was performed on an Novaseq 6000 platform to create 150 bp paired-end reads. Differentially expressed genes (DEG) between the control and P2 groups were identified using the DESeq R package (v1.10.1), with the criteria of |log2(fold change)| ≥ 0.5 and *P*-value < 0.05. These DEGs were used for subsequent analyses.

### Statistical analysis

In our study, we used a completely randomized design with 20 roosters equally allocated into four groups (n = 5 per group), including one control and three different doses of *Pediococcus acidilactici* supplementation. For statistical analysis, we applied a fixed-effects model using one-way ANOVA to evaluate differences among treatment groups. Linear and quadratic regression analyses were used to assess the dose-response relationship. Multiple comparisons were conducted using Duncan’s multiple range test. All analyses were performed using SPSS 25.0 software (SPSS Inc., Chicago, IL, USA). Data are presented as means ± SEM, and statistical significance was considered at *P-value* < 0.05.

## Results

### Effect of different levels of Pediococcus acidilactici on sperm motility and sperm membrane integrity in roosters

**Fig. 1:**
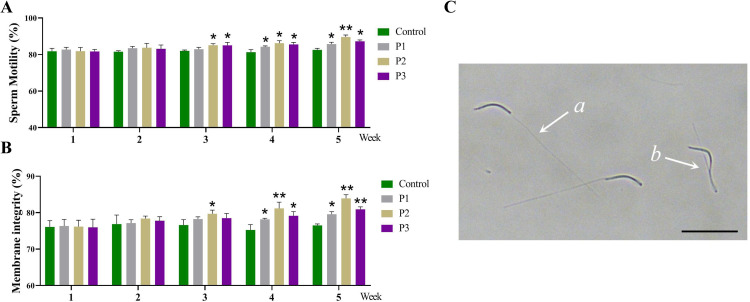
Effect of different levels of *Pediococcus acidilactici* on sperm motility and sperm membrane integrity **in roosters.** (A) Sperm motility assessment. (B and C) Sperm membrane integrity assessment, a: Sperm with uncoiled tails indicates plasma membrane damage; b: Sperm with coiled tails indicates intact plasma membranes, scale bar: 50 µm. Control, the control group fed a basic diet; P1, the treatment group fed a basic diet supplemented with *Pediococcus acidilactici* at 1 × 10^8^ CFU/mL; P2, the treatment group fed a basic diet supplemented with *Pediococcus acidilactici* at 1 × 10^9^ CFU/mL; P3, the treatment group fed a basic diet supplemented with *Pediococcus acidilactici* at 1 × 10^10^ CFU/mL. Data are presented as Mean ± SEM. * *P* < 0.05, ** *P* < 0.01.

As shown in Figs. 1A–C, there were no significant differences in sperm motility or plasma membrane integrity among the groups during weeks 1 and 2 (*P* > 0.05). However, from week 3 onward, sperm motility improved significantly in the P2 and P3 groups compared to the control (*P* < 0.05). Additionally, the P2 group exhibited a significant increase in sperm membrane integrity relative to the control (*P* < 0.05). By week 5, all treatment groups demonstrated significantly higher sperm motility and membrane integrity than the control (*P* < 0.05), with the P2 group showing the most substantial improvements (*P* < 0.01).

### Different levels of Pediococcus acidilactici enhance the motility parameters of rooster sperm

As shown in Table 3, sperm motility parameters improved with longer feeding duration and higher levels of *Pediococcus acidilactici* supplementation compared to the control group. This effect became significant in weeks 4 and 5 (*P* < 0.05), with the P2 and P3 groups showing the most substantial improvements.

**Table 3:** Effect of different levels of *Pediococcus acidilactici* on motility parameters of rooster sperm assessed by CASA.

Item^d^	Time Week	Treatment^e^				SEM^f^	*P*-Value	
		Control	P1	P2	P3		Linear	Quadratic
VAP (µm/s)	1	88.94	87.83	88.97	88.50	2.02	0.480	0.292
	2	89.30	87.64	87.88	90.29	2.32	0.347	0.170
	3	87.75	87.93	88.66	90.95	2.13	0.680	0.409
	4	88.37^b^	90.13^b^	91.89^a^	90.58^a^	1.60	< 0.05	0.296
	5	89.09^c^	90.37^b^^,^^c^	92.68^a^	90.72^a^^,^^b^	1.67	< 0.05	0.118
VSL (µm/s)	1	70.19	69.21	69.15	70.26	1.06	0.091	0.89
	2	70.78	69.81	70.29	70.28	3.07	0.985	0.887
	3	69.32	70.57	71.61	71.55	3.73	0.938	0.758
	4	69.33^b^	71.75^b^	73.18^a^	71.55^b^	1.85	0.156	< 0.05
	5	69.44^c^	71.90^b^	73.34^a^	71.95^a^^,^^b^	1.60	< 0.01	0.374
VCL (µm/s)	1	123.11	121.40	120.85	121.56	1.36	0.284	0.979
	2	123.10	122.75	120.86	123.22	2.44	0.497	0.419
	3	122.64	123.56	124.61	124.21	1.45	0.678	0.680
	4	122.85^b^	124.72^b^	126.64^a^	125.00^b^	1.87	< 0.05	0.069
	5	123.06^b^^,^^c^	124.78^b^	126.79^a^	125.74^b^	1.67	0.076	< 0.05
LIN (%)	1	58.12	57.01	56.87	56.14	1.19	0.824	0.596
	2	58.03	58.17	57.81	57.69	1.53	0.859	0.794
	3	58.17	58.57	59.78	59.75	1.98	0.921	0.720
	4	58.70^c^	59.69^c^	62.65^a^	60.16^b^^,^^c^	2.26	< 0.01	0.020
	5	59.32^c^	60.48^c^	63.93^a^	61.40^b^^,^^c^	2.07	0.017	< 0.05
STR (%)	1	79.00	79.83	77.75	79.90	1.84	0.335	0.180
	2	80.41	79.72	79.54	80.87	2.29	0.797	0.885
	3	79.65^b^	80.68^b^	81.38^b^	82.83^a^	1.75	< 0.05	0.714
	4	79.36^c^	80.69^b^^c^	82.99	82.06^b^	1.64	< 0.01	0.073
	5	79.42^b^	81.51^a^^b^	83.58^a^	83.36^a^	4.33	< 0.05	0.113

a Means bearing different superscripts in the same row differ significantly (*P*-value < 0.05).

b Means bearing different superscripts in the same row differ significantly (*P*-value < 0.05).

c Means bearing different superscripts in the same row differ significantly (*P*-value < 0.05).

d VAP, Average Path Velocity; VSL, Average Straight-Line Velocity; VCL, Average Curvilinear Velocity; LIN, Linearity Index [(VSL/VCL) × 100]; STR, Straightness Index [(VSL/VAP) × 100].

e Control, the control group fed a basic diet; P1, the treatment group fed a basic diet supplemented with *Pediococcus acidilactici* at 1 × 10^8^ CFU/mL; P2, the treatment group fed a basic diet supplemented with *Pediococcus acidilactici* at 1 × 10^9^ CFU/mL; P3, the treatment group fed a basic diet supplemented with *Pediococcus acidilactici* at 1 × 10^10^ CFU/mL.

f SEM standard error of the means.

### Different levels of Pediococcus acidilactici enhance the viability and mitochondrial membrane potential of rooster sperm

**Fig. 2:**
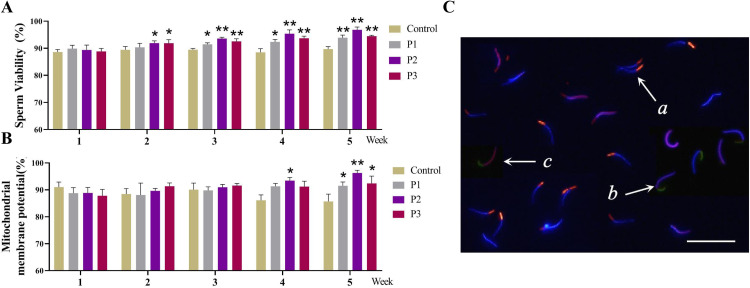
Effect of different levels of *Pediococcus acidilactici* on sperm viability and mitochondrial membrane potential in roosters. (A) Sperm viability analysis. (B) Sperm mitochondrial membrane potential Analysis. (C) Sperm Hoechst 33342, PI, and JC-1 combined fluorescent staining, a: Live sperm cells (Hoechst 33342 positive and PI negative), and mitochondrial membranes, exhibit blue fluorescence at the head and intense orange-red fluorescence at the midpiece, with a high mitochondrial transmembrane potential; b: Live sperm cells and low mitochondrial transmembrane potential show blue fluorescence at the head and green at the midpiece. c: dead sperm cells show red fluorescence at the head and less or no green fluorescence at the midpiece. Scale bar: 50 μm. Control, the control group fed a basic diet; P1, the treatment group fed a basic diet supplemented with *Pediococcus acidilactici* at 1 × 10^8^ CFU/mL; P2, the treatment group fed a basic diet supplemented with *Pediococcus acidilactici* at 1 × 10^9^ CFU/mL; P3, the treatment group fed a basic diet supplemented with *Pediococcus acidilactici* at 1 × 10^10^ CFU/mL. Data are presented as Mean ± SEM. * *P* < 0.05, ** *P* < 0.01.

Mitochondrial membrane potential is significantly correlated with sperm motility parameters, including VCL, VSL, and VAP. Sperm with higher mitochondrial activity typically exhibit higher fertilization rates during in vitro fertilization (Marchetti et al., 2004; Ren et al., 2018). As shown in Fig. 2A–C, with the extension of the feeding time, the sperm viability of the treatment group supplemented with *Pediococcus acidilactici* showed a significant upward trend (*P* < 0.05). Moreover, a significant difference was observed between the treatment and control groups at week 2 (*P* < 0.05). Meanwhile, the sperm mitochondrial membrane potential also showed significant differences at weeks 4 and 5 (*P* < 0.05). Notably, the P2 treatment group showed a highly significant improvement in sperm viability and mitochondrial membrane potential (*P* < 0.01).

### Different levels of Pediococcus acidilactici reduce apoptosis and protamine deficiency in rooster sperm

**Fig. 3:**
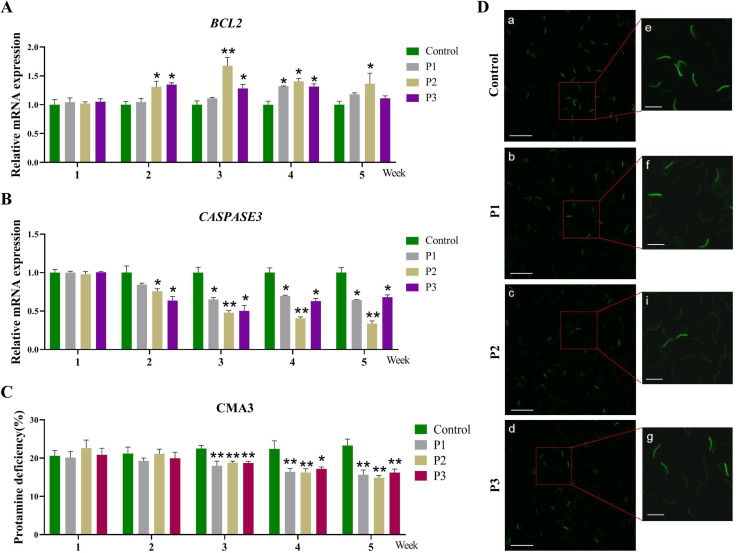
**Effect of different levels of *Pediococcus acidilactici* on sperm apoptosis and protamine deficiency in roosters.** (A and B) mRNA expression levels of apoptosis-related genes *Bcl2* and *Caspase3* in sperm. (C and D) Detection of sperm protamine deficiency (DNA integrity) using CMA3 staining, bright green fluorescence indicates DNA-damaged sperm, while dim green fluorescence represents normal sperm. Scale: (a-d) 100 µm, (e-g) 25 µm. Control, the control group fed a basic diet; P1, the treatment group fed a basic diet supplemented with *Pediococcus acidilactici* at 1 × 10^8^ CFU/mL; P2, the treatment group fed a basic diet supplemented with *Pediococcus acidilactici* at 1 × 10^9^ CFU/mL; P3, the treatment group fed a basic diet supplemented with *Pediococcus acidilactici* at 1 × 10^10^ CFU/mL. Data are presented as Mean ± SEM. * *P* < 0.05, ** *P* < 0.01.

Protamine deficiency is commonly used as an indicator for assessing DNA integrity in sperm nuclei (Sanovec et al., 2024). The effect of *Pediococcus acidilactici* supplementation on sperm apoptosis and protamine levels is presented in Fig. 3A and B. Compared to the control group, the expression of the anti-apoptotic gene *BCL2* was significantly upregulated (*P* < 0.05), whereas the expression of *Caspase3* was significantly downregulated (*P* < 0.05) from week 2 onward. This trend persisted through week 5, suggesting a sustained protective effect of *Pediococcus acidilactici* on sperm viability. Similarly, with the increasing duration of *Pediococcus acidilactici* supplementation, the level of sperm protamine deficiency gradually decreased (Fig. 3C and D). Notably, from weeks 3 to 5, all treatment groups supplemented with *Pediococcus acidilactici* exhibited significantly lower protamine deficiency compared to the control group (*P* < 0.01). These findings suggest that *Pediococcus acidilactici* supplementation significantly improves sperm apoptosis and protamine levels, thereby exerting a positive impact on sperm DNA integrity.

### Effect of different levels of Pediococcus acidilactici on splasma reproductive hormone levels of roosters

Testosterone is a key hormone related to spermatogenesis and metabolism, whose secretion is stimulated by FSH and LH. Generally, LH activates the protein kinase A (PKA) pathway and promotes the expression of key steroid genes by stimulating G proteins and adenylyl cyclase (Gao et al., 2022). Table 4 presents the effects of *Pediococcus acidilactici* supplementation on serum reproductive hormone levels. The study found that by week 5, these hormone levels showed significant differences (*P* < 0.05) compared to the control group, with the highest expression observed in the P2 treatment group. These findings suggest that *Pediococcus acidilactici* supplementation may positively regulate reproductive hormones, thereby influencing sperm production and metabolism.

**Table 4:** Effect of different levels of *Pediococcus acidilactici* on splasma reproductive hormone levels of roosters.

Item^d^	Time Week	Dietary treatment^e^				SEM^f^	*P*-Value	
		Control	P1	P2	P3		Linear	Quadratic
T (ng/mL)	5	1.3^b^	1.69^b^	2.31^a^	1.16^b^	0.8	< 0.01	0.101
FSH (mIU/mL)	5	2^c^	2.86^b^^,^^c^	3.93^a^	3.9^a^^b^	0.88	< 0.01	0.061
LH (mIU/mL)	5	5.98^c^	6.63^b^^,^^c^	9.12^a^	8.93^a^	1.56	< 0.0001	< 0.05

a Means bearing different superscripts in the same row differ significantly (*P*-value < 0.05).

b Means bearing different superscripts in the same row differ significantly (*P*-value < 0.05).

c Means bearing different superscripts in the same row differ significantly (*P*-value < 0.05).

d T testosterone, FSH follicle-stimulating hormone, E2 estradiol.

e Control, the control group fed a basic diet; P1, the treatment group fed a basic diet supplemented with *Pediococcus acidilactici* at 1 × 10^8^ CFU/mL; P2, the treatment group fed a basic diet supplemented with *Pediococcus acidilactici* at 1 × 10^9^ CFU/mL; P3, the treatment group fed a basic diet supplemented with *Pediococcus acidilactici* at 1 × 10^10^ CFU/mL.

f SEM standard error of the means.

### Effect of different levels of Pediococcus acidilactici on the antioxidant capacity of testes

Table 5 presents the effects of different levels of *Pediococcus acidilactici* supplementation on testicular antioxidant capacity in breeding roosters at week 5. Compared to the control group, the activities of T-SOD, GSH-Px, and T-AOC were significantly increased in the P2 and P3 groups (*P* < 0.01), while MDA levels were significantly reduced (*P* < 0.01). Moreover, a significant linear dose-response relationship was observed (*P* < 0.01).

**Table 5:** Effect of different levels of *Pediococcus acidilactici* on the antioxidant capacity of testes.

Item^d^	Time Week	Dietary treatment^e^				SEM^f^	*P*-Value	
		Control	P1	P2	P3		Linear	Quadratic
T-SOD (U/mg prot)	5	77.25^c^	81.90^b^	86.15^a^	87.13^a^	4.46	< 0.001	0.069
MDA (nmoL/mg prot)	5	2.13^a^	1.34^a^^,^^b^	0.73^c^	1.05^b^^,^^c^	0.76	< 0.01	0.054
GSH-Px (U/mg prot)	5	103.52^b^	104.57^b^	162.94^a^	161.91^a^	28.93	< 0.001	0.265
T-AOC (nmoL/mg prot)	5	0.75^c^	1.05^b^^c^	2.02^a^	1.86^a^^b^	0.76	< 0.01	0.417

a Means bearing different superscripts in the same row differ significantly (*P*-value < 0.05).

b Means bearing different superscripts in the same row differ significantly (*P*-value < 0.05).

c Means bearing different superscripts in the same row differ significantly (*P*-value < 0.05).

d T-SOD superoxide dismutase, MDA malondialdehyde content, GSH-Px glutathione peroxidase, T-AOC total antioxidant capacity.

e Control, the control group fed a basic diet; P1, the treatment group fed a basic diet supplemented with *Pediococcus acidilactici* at 1 × 10^8^ CFU/mL; P2, the treatment group fed a basic diet supplemented with *Pediococcus acidilactici* at 1 × 10^9^ CFU/mL; P3, the treatment group fed a basic diet supplemented with *Pediococcus acidilactici* at 1 × 10^10^ CFU/mL.

f SEM standard error of the means.

### Effect of different levels of Pediococcus acidilactici on histological parameters of roosters’ testes

**Fig. 4:**
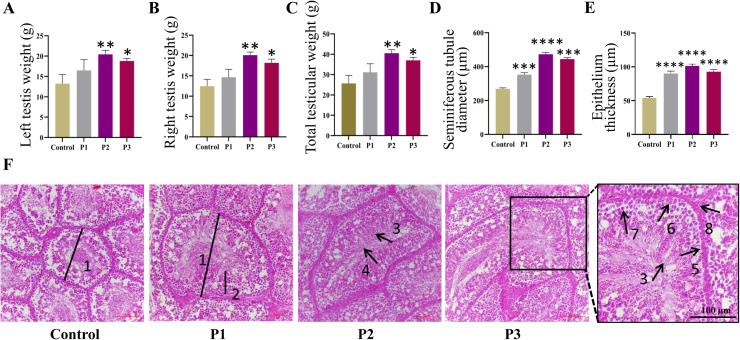
**Effects of different levels of *Pediococcus acidilactici* on testicular histological parameters of roosters.** (A-C) Testicular weight. (D) Diameter of seminiferous tubules in the testis. (E) Epithelium thickness. (F) Hematoxylin and eosin (HE) staining of testicular tissue. 1, Seminiferous tubule diameter; 2, Seminiferous tubule epithelial height; 3, Spermatozoon; 4, Spermatid; 5, Spermatogonia; 6, Primary spermatocyte; 7, Secondary spermatocyte; 8, Leydig cell. Scale: 100 µm. Control, the control group fed a basic diet; P1, the treatment group fed a basic diet supplemented with *Pediococcus acidilactici* at 1 × 10^8^ CFU/mL; P2, the treatment group fed a basic diet supplemented with *Pediococcus acidilactici* at 1 × 10^9^ CFU/mL; P3, the treatment group fed a basic diet supplemented with *Pediococcus acidilactici* at 1 × 10^10^ CFU/mL.

At week 5, testicular tissues were collected for weighing and HE staining. As shown in Fig. 4A–F, testicular weight, seminiferous tubule diameter and seminiferous epithelial thickness increased with *Pediococcus acidilactici* supplementation, with the most significant effects observed in the P2 treatment group (*P* < 0.05). These findings suggest that *Pediococcus acidilactici* supplementation may promote testicular development and function by improving the physiological state of the reproductive system.

### Effect of different levels of Pediococcus acidilactici on the transcriptional profile of testicular tissues

**Fig. 5:**
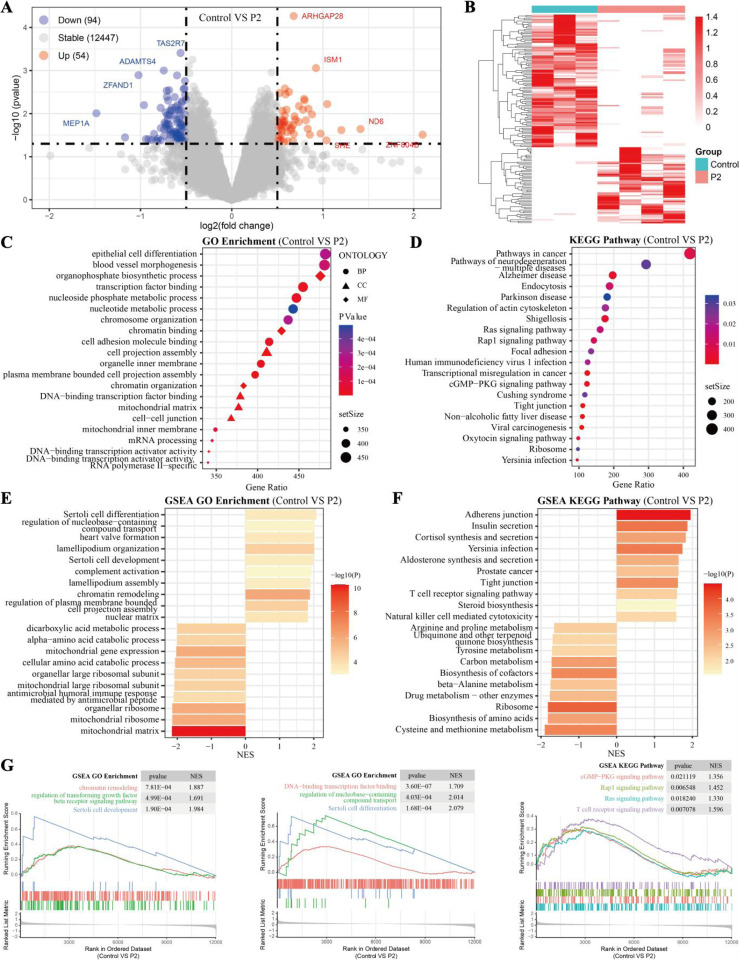
**Transcriptome analysis of testicular tissue in roosters after 5 weeks of feeding.** (A and B) Volcano plot and hierarchical clustering analysis showing 94 downregulated and 54 upregulated mRNAs. (C and D) GO and KEGG enrichment analyses of differentially expressed mRNAs. (E and F) GSEA results of GO and KEGG pathways, showing the top 10 upregulated and downregulated pathways. (G) GSEA for gene sets related to testis development expression. Control (n=3), the control group fed a basic diet; P2 (n=4), the treatment group fed a basic diet supplemented with *Pediococcus acidilactici* at 1 × 10^9^ CFU/mL.

To explore the effects of *Pediococcus acidilactici* on testicular gene expression, RNA-seq was performed on testicular samples from the control and P2 groups at the end of week 5. As shown in Fig. 5A and 5B, transcriptomic analysis identified 54 upregulated and 94 downregulated differentially DEGs in the P2 group. GO analysis revealed that most DEGs were significantly enriched in biological processes, with *Pediococcus acidilactici* affecting catalytic activity and cellular processes, as shown in Fig. 5C. The GO functional enrichment analysis further indicated that these DEGs were significantly involved in epithelial cell differentiation, transcription factor binding, cell adhesion molecule binding, and other related processes. Additionally, KEGG pathway enrichment analysis, as shown in Fig. 5D, demonstrated that the identified DEGs participated in multiple signaling pathways, including endocytosis, regulation of the actin cytoskeleton, Ras, Rap1, cGMP-PKG, and ribosome pathways. Furthermore, GSEA analysis revealed that in the P2 group, pathways related to Sertoli cell differentiation, regulation of nucleobase-containing compound transport, Sertoli cell development, T cell receptor signaling pathway, and steroid biosynthesis were upregulated, as shown in Fig. 5E and 5F. In contrast, pathways associated with the mitochondrial matrix, ribosome, and biosynthesis of amino acids were downregulated. Additionally, several classical pathways related to testicular cell development and function, such as the Ras, Rap1, and cGMP-PKG signaling pathways, were significantly upregulated in the P2 group, as shown in Fig. 5G.

**Fig. 6:**
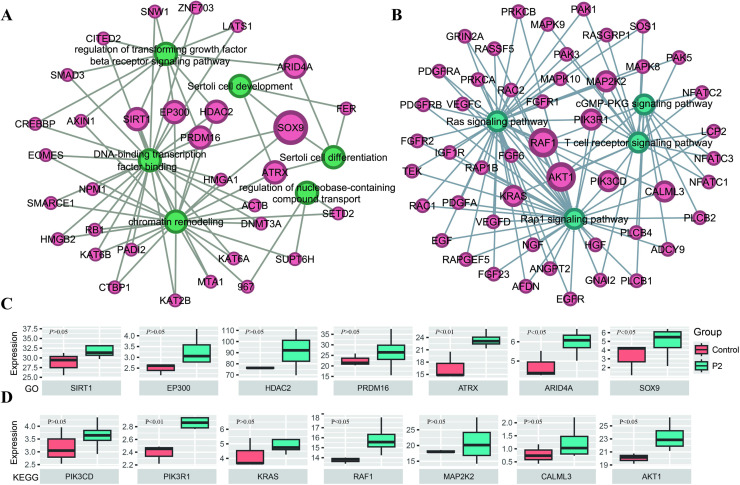
**GO and KEGG pathway enrichment analyses.** (A) Network of the enriched GO terms for the mRNA targets. (B) Network of the enriched KEGG terms for the mRNA targets. (C and D) The significant differential distribution of genes associated with cell differentiation and chromatin remodeling in roosters testes. Control (n=3), the control group fed a basic diet; P2 (n=4), the treatment group fed a basic diet supplemented with *Pediococcus acidilactici* at 1 × 10^9^ CFU/mL.

Additionally, we selected key GO-enriched terms and KEGG pathways related to testicular development and function (Fig. 6A and 6B) and constructed network diagrams. Through these networks, we identified a series of genes that may play crucial roles in testicular development and function. GO analysis revealed significant enrichment of biological processes associated with cell differentiation, transcriptional regulation, and chromatin remodeling (Fig. 6A). Notably, genes involved in spermatogonia differentiation, such as SOX9 and FER, as well as genes associated with DNA-binding transcription factor activity, such as EOMES and NFATC1, exhibited strong connectivity within the network. In addition, genes involved in chromatin remodeling, such as HMGB2 and KAT6B, also occupied key positions in the network. KEGG pathway analysis further highlighted signaling pathways that play crucial roles in cellular signal transduction, including the Ras and Rap1 signaling pathways (Fig. 6B). Genes in these pathways, such as FGFR1, RAF1, and AKT1, exhibited centrality within the network, suggesting that they may play a core role in testicular development and function. Furthermore, we observed that in the P2 group, the expression levels of several genes related to cell differentiation and chromatin remodeling, including RAF1, PIK3R1, ATRX, ARID4A, and SOX9, were significantly higher than in the control group (Fig. 6C and 6D). These results indicate that *Pediococcus acidilactici* supplementation may influence testicular development and function in roosters by regulating the expression of these key genes.

## Discussion

This study comprehensively evaluated the effects of dietary supplementation with *Pediococcus acidilactici* on rooster reproductive performance, with a focus on sperm quality, testicular development, and transcriptomic changes. The results demonstrated that at a concentration of 1 × 10^9^ CFU/mL, *Pediococcus acidilactici* significantly enhanced sperm motility, viability, mitochondrial function, and testicular antioxidant and histological parameters, while reducing apoptosis and protamine deficiency. Furthermore, transcriptomic analysis provided key insights into the gene expression and pathways regulated by this probiotic. These findings offer valuable evidence for the potential role of probiotics in improving rooster fertility.

This study observed improvements in sperm motility and kinematic parameters, highlighting the potential value of *Pediococcus acidilactici* in enhancing male fertility in poultry. The experimental results showed a significant increase in mitochondrial membrane potential in the treatment group, indicating improved mitochondrial function. Given that the energy required for sperm motility is primarily supplied by mitochondria located in the midpiece of the sperm neck, this functional enhancement is crucial for maintaining sperm motility. Mitochondria generate energy for sperm movement through phosphorylation of flagellar proteins (Sakkas et al., 2003; Turner, 2006; Urner and Sakkas, 2003), thereby facilitating normal fertilization. Therefore, the improvement in mitochondrial function may be a key factor contributing to the significant enhancement of sperm motility parameters, such as VCL, VSL, and LIN, observed in this study. This finding is consistent with the study by Lee EH et al., which demonstrated that probiotics improve sperm motility and kinematics in mice while enhancing mitochondrial function in mature sperm (Lee et al., 2024). In addition, this study demonstrated a significant improvement in sperm plasma membrane integrity in the P2 and P3 treatment groups. As a critical indicator of sperm function and fertilization capacity, an intact plasma membrane is essential for maintaining cellular homeostasis, regulating ion exchange, and facilitating signal transduction (Flesch and Gadella, 2000). Therefore, the enhancement of mitochondrial function and the improvement of sperm plasma membrane integrity may have a synergistic effect, collectively supporting sustained sperm motility and maximizing their potential to reach the oocyte. These findings further illustrate the beneficial effects of *Pediococcus acidilactici* supplementation on multiple key factors of sperm function, providing strong evidence for its potential application in improving male fertility.

The dense and stable nuclear structure of mature mammalian sperm is maintained by protamines (Lolis et al., 1996). Protamines are basic proteins that facilitate DNA condensation by packaging the genomic DNA into the sperm head, a process essential for the formation of functional sperm (Oliva, 2006). The formation of inter- and intramolecular disulfide bonds within sperm protamines induces chromatin condensation and alters sperm morphology, thereby promoting sperm maturation, differentiation, and nuclear compaction, ultimately ensuring the normal morphology of the sperm head (Hecht, 1998). Moreover, abnormal sperm morphology is associated with nuclear chromatin integrity and infertility (Dehghanpour et al., 2017). Sperm DNA damage has been shown to negatively affect fertilization, implantation, embryonic development, and even offspring health (Aitken and De Iuliis, 2007; Bungum et al., 2012), highlighting DNA damage as a crucial factor influencing sperm quality. In this study, after three weeks of *Pediococcus acidilactici* supplementation, all treatment groups exhibited a significant reduction in protamine deficiency levels, along with a marked decrease in sperm apoptosis. These findings are consistent with previous studies by Talebi AR et al. (Talebi et al., 2016) and Dehghanpour F et al. (Dehghanpour et al., 2017). Furthermore, apoptosis has been identified as a major contributor to sperm DNA strand breaks (Virro et al., 2004), suggesting that the observed reduction in apoptosis may play a crucial role in preserving sperm DNA integrity.

The testes, as the principal reproductive organs, are responsible for both spermatogenesis and T production. Spermatogenesis is regulated by multiple gonadal hormones (Beattie et al., 2015). FSH and LH secreted by the pituitary gland, along with T produced by Leydig cells, are critical modulators of spermatogenesis. Testosterone, a 19-carbon steroid hormone, is released in response to FSH and LH stimulation and serves as the primary hormonal regulator. It directly promotes testicular development, stimulates the proliferation of spermatogonia, and plays a pivotal role in male spermatogenesis, sperm maturation, and the development of secondary sexual characteristics (Heng et al., 2017). A deficiency in testosterone is a direct cause of spermatogenic impairment. Consequently, testosterone is considered an essential hormone for spermatogenesis, particularly in older roosters, where testosterone levels tend to be lower. In this study, we observed significant differences in serum T, FSH, and LH levels between the P2 and P3 treatment groups and the control group at week 5. Similar improvements in T, FSH, and LH levels have been reported following flaxseed oil supplementation in aging roosters (Qi et al., 2019). Testicular size is a critical parameter for assessing male reproductive function, as it serves as an indicator of reproductive abnormalities and provides insights into sperm production capacity (Amann, 1999; Donoghue et al., 2003). Consistent with the well-documented phenomenon of testicular asymmetry in avian species (Abdul-Rahman et al., 2018), we observed that, following five weeks of *Pediococcus acidilactici* supplementation, the right testis was approximately 5 % smaller than the left. Additionally, testicular weight in both the P2 and P3 treatment groups was significantly greater than in the control group. Furthermore, we observed a significant difference in the diameter of the seminiferous tubules between groups, indicating a potential beneficial effect of *Pediococcus acidilactici* on testicular morphology and spermatogenesis.

Research has demonstrated that antioxidants, both enzymatic and non-enzymatic, can enhance the antioxidant defense system and play a pivotal role in disease prevention and treatment (Seifried et al., 2007). A previous in vitro study provided preliminary evidence indicating that probiotics can effectively protect sperm from lipid peroxidation, thereby preserving sperm motility and viability (Helli et al., 2022). Moreover, aging in male individuals is often accompanied by elevated oxidative stress levels in the testes, primarily driven by increased reactive oxygen species (ROS). This elevated oxidative stress has a detrimental effect on the seminiferous epithelium, leading to spermatogenic dysfunction and reduced sperm production (Selvaratnam and Robaire, 2016). Previous studies have also shown that probiotic supplementation can restore the activity of antioxidant enzymes, such as SOD and GSH-Px, in both the serum and sperm of obese rats. Concurrently, probiotics have been found to reduce levels of MDA and nitric oxide (NO), highlighting the antioxidant properties of probiotics and their potential to mitigate oxidative damage to a certain extent (Abbasi et al., 2021). These findings further underscore the potential role of probiotics in combating oxidative stress and improving male reproductive health. The results of this study are consistent with previous findings, as supplementation with *Pediococcus acidilactici* for 5 weeks significantly increased the levels of SOD and GSH-Px in testicular tissue, while reducing MDA levels. Furthermore, T-AOC was significantly improved. In addition, existing studies have confirmed that oxidative stress can lead to DNA damage and accelerate the cell division process, ultimately affecting sperm count (Sumner et al., 2019). Therefore, the observed improvement in DNA integrity in this study may be closely related to the ability of *Pediococcus acidilactici* to alleviate oxidative stress damage through its antioxidant effects.

Spermatogenesis is a highly complex process requiring precise regulation of multiple genes. GO classification analysis of the testicular transcriptome in roosters treated with *Pediococcus acidilactici* revealed that DEGs were predominantly enriched in functional categories related to cell cycle, mitosis, cellular processes, and catalytic activity. This enrichment aligns with the fundamental biological events of spermatogenesis, including the mitotic proliferation of spermatogonia, meiotic division of primary spermatocytes, and their differentiation into round spermatids (Asano and Tajima, 2017). Further GSEA analysis identified key genes influenced by *Pediococcus acidilactici*, including SOX9, FGFR1, RAF1, and AKT1, along with critical signaling pathways such as the Ras, cGMP-PKG, and chromatin remodeling, all of which are likely to play pivotal roles in the regulation of testicular function and spermatogenesis (Gianzo and Subirán, 2020). Notably, SOX9 is essential for Sertoli cell differentiation and germ cell development (Ortega et al., 2015) while AKT1 has been implicated in maintaining normal testicular physiology by modulating redox homeostasis (Kim et al., 2012). Moreover, GO and KEGG enrichment analyses demonstrated a significant upregulation of pathways associated with cell differentiation and chromatin remodeling, suggesting that *Pediococcus acidilactici* may enhance spermatogenesis by optimizing the cellular and molecular microenvironment necessary for this process. These findings provide new insights into the potential mechanisms by which *Pediococcus acidilactici* influences testicular function and reproductive health.

## Conclusion

In summary, this study demonstrates that dietary supplementation with *Pediococcus acidilactici* significantly enhances rooster reproductive performance by improving sperm quality, promoting testicular development, and modulating key molecular pathways. These findings establish a scientific basis for reproductive management in poultry production and offer important insights into the potential application of probiotics for optimizing reproductive performance.

## Declaration of competing interest

The authors declare that they have no known competing financial interests or personal relationships that could have appeared to influence the work reported in this paper.
